# Prostate cancer detection in routine clinical practice: a large cohort study in Japan

**DOI:** 10.1038/s41598-026-51230-6

**Published:** 2026-05-02

**Authors:** Akinori Wada, Yuki Okinaka, Masayuki Nagasawa, Toru Yoshida, Satoshi Ishitoya, Takashi Osafune, Chul Jang Kim, So Ushijima, Ryosuke Murai, Mitsuhiro Narita, Zenkai Nishikawa, Hiroki Soga, Hiroshi Ushida, Yuji Sakano, Yoshio Naya, Yoshimasa Harada, Taichi Sano, Teruhiko Tsuru, Kenichi Kobayashi, Kazuaki Yamanaka, Tetsuya Yoshida, Susumu Kageyama

**Affiliations:** 1 Department of Urology, Shiga University of Medical Science, Seta Tsukinowa-cho, Otsu, Shiga 520-2192 Japan; 2 Department of Urology, Yasu City Hospital, Yasu, Shiga Japan; 3 Shiga General Hospital, Moriyama, Shiga Japan; 4 Japanese Red Cross Otsu Hospital, Otsu, Shiga Japan; 5 Otsu City Hospital, Otsu, Shiga Japan; 6 Kohka Public Hospital, Koka, Shiga Japan; 7 Omihachiman Community Medical Center, Omihachiman, Shiga Japan; 8 Nagahama City Hospital, Nagahama, Shiga Japan; 9 Omi Medical Center, Kusatsu, Shiga Japan; 10 Hino Memorial Hospital, Hino, Shiga Japan; 11 Toyosato Hospital, Toyosato, Shiga Japan; 12 JCHO Shiga Hospital, Otsu, Shiga Japan; 13 National Hospital Organization Higashi-ohmi General Medical Center, Higashiomi, Shiga Japan; 14 Nagahama City Kohoku Hospital, Nagahama, Shiga Japan; 15 Nagahama Red Cross Hospital, Nagahama, Shiga Japan; 16 Hikone Municipal Hospital, Hikone, Shiga Japan; 17 Takashima Municipal Hospital, Takashima, Shiga Japan

**Keywords:** PSA screening, Prostate cancer, Real world data, Primary therapy, Risk classification, Cancer, Diseases, Oncology, Urology

## Abstract

Population-based organized prostate-specific antigen (PSA) screening is implemented in 80% of Japanese municipalities; however, Shiga Prefecture remains a unique exception without such a systematic program. This study characterized the longitudinal clinical features and treatment patterns in this opportunistic testing environment using data from 1716 patients diagnosed via prostate biopsy in 2012, 2017, and 2022. While median PSA levels remained stable (10.40–11.43 ng/mL), median age at diagnosis increased from 72 to 74 years. Over the decade, the incidence of International Society of Urological Pathology Grade Group 1 and cT1c stages decreased significantly (*p* < 0.001), with nearly 90% of cases being cT2 or higher in 2022. Risk classification showed a decrease in low-risk cases and a rise in high-risk cases. Regarding treatment, radical prostatectomy rates remained stable at approximately 25%, whereas the overall use of active surveillance (AS) increased from 1 to 9%. Notably, among low-risk patients, AS adoption rose markedly from 2.3% in 2012 to 68% in 2022. While clinical practices have evolved to successfully minimize unnecessary invasive intervention, these findings suggest that clinical progress alone cannot fully compensate for the lack of organized efforts to improve early detection.

## Introduction

Prostate-specific antigen (PSA) testing is a valuable tool for the early detection of prostate cancer^1,2^. However, its impact on reducing mortality remains controversial. Large randomized controlled trials, such as the European Randomized Study of Screening for Prostate Cancer (ERSPC), reported a reduction in disease-specific mortality^3,4^, whereas the Prostate, Lung, Colorectal, and Ovarian (PLCO) and Cluster Randomized Trial of PSA Testing for Prostate Cancer (CAP) trials initially showed no such benefit^5–7^. The PLCO results have been questioned due to high screening rates in the control group^8–10^, and long-term follow-up of the CAP trial recently suggested a reduction in deaths at 15 years^11^. Furthermore, the ERSPC and the ERSPC Rotterdam Study Group reported that increased screening over time led to lower mortality rates and a reduced risk of overdiagnosis^4,12^. Despite potential benefits, PSA screening carries risks of overdiagnosis and overtreatment^13–15^. Consequently, international guidelines recommend shared decision-making after a thorough discussion of risks and benefits^16,17^, and the debate over organized PSA screening continues.

In Japan, prostate cancer incidence and mortality are rising, with approximately 88,000 new cases and 13,000 deaths in 2020^18^. While nearly 80% of Japanese municipalities implement population-based organized screening^19^, Shiga Prefecture (population: approximately 1.4 million) presents a unique exception. Organized screening was only briefly conducted in one city and ceased entirely by 2017^20^. This shift occurred primarily because the Japanese government, citing insufficient evidence regarding cost-effectiveness and a mortality benefit, does not currently include PSA screening in its national recommendations for population-based program. This environment provides a rare opportunity to evaluate the “real-world” impact of opportunistic testing and routine clinical care alone, virtually unbiased by the effects of an organized program.

Given the global debate over organized versus opportunistic screening, analyzing a region that relies solely on clinical practice is essential to understanding whether modern diagnostic pathways and guideline-based treatments can sufficiently manage prostate cancer outcomes. It should be noted that this study is not a clinical trial designed to evaluate the efficacy of screening on mortality, but rather a descriptive, longitudinal observational study aimed at documenting the clinical status in a specific Japanese prefecture. Considering these regional characteristics, we sought to determine whether current clinical practices alone are sufficient for early detection while successfully minimizing unnecessary clinical intervention for low-risk disease. By evaluating clinical features and treatment patterns over a decade, we aimed to evaluate the long-term impact and limitations of opportunistic PSA testing in a real-world setting.

## Patients and methods

### Patients

This multicenter observational study was conducted in Shiga Prefecture, Japan. We included patients who underwent prostate biopsy following an elevated PSA and were subsequently diagnosed with prostatic adenocarcinoma in 2012, 2017, and 2022. Cases with other histological types, including neuroendocrine tumors and non-epithelial tumors, were excluded. Clinical T1a-b prostate cancers incidentally detected during surgery for benign prostatic hyperplasia were also excluded. Data were collected from 14, 14, and 17 institutions in 2012, 2017, and 2022, respectively.

### Data collection and definitions

Patients’ clinicopathological data were extracted from their medical records. For this study, we collected information on age, initial PSA values, reasons for PSA measurement, International Society of Urological Pathology (ISUP) Grade Groups based on biopsy Gleason scores, clinical stage (TNM classification), and primary treatment selection. To estimate the representativeness of our cohort, the coverage rate was calculated by dividing the number of patients in this study by the total number of new prostate cancer diagnoses reported in the Shiga Prefectural Government Cancer Registry for the corresponding years.

### PSA testing categories

The reasons for PSA measurement were categorized into seven groups as previously reported^21^: (1) testing in general practice clinics (no urologist involvement), (2) testing in urologic clinics (incidentally diagnosed), (3) repeat measurement due to previously elevated PSA (PSA monitoring), (4) organized screening (population-based screening), (5) health check-up (individual active measurement), (6) evaluation for metastatic disease of unknown origin, and (7) other reasons. This categorization, while specific to our study design, was developed to reflect the diverse clinical pathways through which PSA is measured in actual practice in Shiga. In this study, “opportunistic PSA testing” was defined as any non-organized measurement (categories 1, 2, 3, 5 and 6), encompassing both physician-initiated and patient-requested tests during routine consultations. Specifically, categories 1 and 2 likely included men who had undergone relatively few prior PSA tests. “Organized screening” (category 4) referred strictly to systematic, municipality-managed programs. We acknowledge that these categories include both symptomatic and asymptomatic testing, which may deviate from strict epidemiological definitions of screening.

### Pathological and risk classification

The threshold PSA level of 4.0 ng/mL was utilized for recommending a prostate biopsy; furthermore, a biopsy was also recommended for patients with abnormal digital rectal examination findings, regardless of their PSA level. Pathological features were classified using the ISUP Grade Group (GG) system based on the Gleason score as follows: GG 1 (Gleason score ≤ 6); GG 2 (3 + 4=7); GG 3 (4 + 3=7); GG 4 (8); and GG 5 (9–10)^22^. Consistent with our previous reports^20,21^, risk classification was based on the criteria defined by Godtman et al.^23^: low risk was defined as T1, not N1 or M1, GG1, and PSA < 10 ng/mL; intermediate risk as T1-2, not N1 or M1, and GG2 or GG3 and/or PSA < 20 ng/mL; high risk as T1-4, not N1 or M1, and GG ≥ 4 and/or PSA < 100 ng/mL; and advanced disease as N1 and/or M1 and/or PSA ≥ 100 ng/mL. Within this framework, “high-grade cancer” was specifically defined as GG 4 or 5. Furthermore, to evaluate the detection of clinically significant tumors, we defined GG ≥ 2 as the threshold for clinically significant prostate cancer^24,25^. This study was approved by the Ethics Committee of Shiga University of Medical Science (Approval number: R2023-046) and by all participating institutions. Informed consent was obtained via an opt-out process on the Shiga University of Medical Science Hospital website, with individuals who opted out excluded from the study. The study was performed in accordance with the Declaration of Helsinki and all relevant guidelines and regulations.

### Statistical analyses

Statistical analyses were conducted using IBM SPSS for Windows, version 29.0 (IBM, Armonk, NY, USA). Group differences were assessed using the Kruskal-Wallis test and chi-square test. Trends in annual changes in clinical T stage and ISUP grade group were evaluated using the Cochran-Armitage test. A two-sided *p* value < 0.05 was considered statistically significant.

## Results

### Patient demographics

A total of 1716 patients with prostate cancer were included in this study. Patient demographics are summarized in Table 1. The number of patients diagnosed in each year was 431, 553, and 732 in 2012, 2017, and 2022, respectively. According to the Shiga Prefectural Government Cancer Registry, 616 and 896 individuals received a first prostate cancer diagnosis in 2012 and 2017, respectively. Although official data for 2022 are not yet available, our cohort captures more than 60% of all newly diagnosed prostate cancer cases in Shiga Prefecture. Since 2016, all hospitals and designated medical institutions have been required to report prostate cancer diagnoses to the Shiga Prefectural Government Cancer Registry. The Death Certificate Only ratio for this registry was 2.8% in 2012 and 0.9% in 2017, reflecting a high level of data accuracy^26^.

**Table 1:** Patient demographics.

Year	2012	2017	2022	*P*-value
Number of patients	431	553	732	
Median age, years	72 (52–90)	73 (44–92)	74 (50–97)	< 0.001
Median initial PSA (ng/mL)	10.92 (3.30–7219)	11.43 (1.15–8684)	10.40 (0.12–15003)	0.613
International society of urological pathology gleason grade				
GG1	100 (23%)	86 (16%)	84 (11%)	< 0.001
GG2	96 (22%)	132 (24%)	147 (20%)	
GG3	84 (19%)	98 (18%)	127 (17%)	
GG4	72 (17%)	79 (14%)	190 (26%)	
GG5	79 (18%)	158 (29%)	184 (25%)	
Clinical T stage				
T1c	145 (34%)	119 (22%)	84 (12%)	< 0.001
T2	173 (40%)	284 (51%)	443 (60%)	
T3	88 (20%)	112 (20%)	156 (21%)	
T4	21 (5%)	35 (6%)	43 (6%)	
Unknown	4 (4%)	3 (1%)	6 (1%)	
N stage				
N0	380 (88%)	480 (87%)	640 (87%)	0.879
N1	48 (11%)	71 (12%)	88 (12%)	
Unknown	3 (1%)	2 (1%)	4 (1%)	
M stage				
M0	379 (88%)	467 (84%)	619 (85%)	0.299
M1	49 (11%)	81 (15%)	109 (14%)	
Unknown	3 (1%)	5 (1%)	4 (1%)	
Risk classification				
Low risk	43 (10%)	29 (5%)	25 (3%)	< 0.001
Intermediate risk	175 (41%)	225 (41%)	255 (35%)	
High risk	128 (30%)	180 (33%)	295 (40%)	
Advanced	82 (19%)	118 (21%)	153 (21%)	
Unknown	3 (1%)	1 (0.1%)	4 (0.5%)	

The median age at diagnosis was 72, 73, and 74 years in 2012, 2017, and 2022, respectively, representing a statistically significant increase over time. Median PSA levels remained stable during this period, ranging from 10.40 to 11.43 ng/mL. The incidence of GG1 cancers decreased significantly from 23% in 2012 to 11% in 2022 (*p* < 0.001), while high-grade cancers increased from 35 to 51% (*p* < 0.001). The proportion of cT1c cancers declined from 34% in 2012 to 12% in 2022 (*p* < 0.001), with nearly 90% of cases classified as cT2 or higher in 2022 (Table 1). Regarding clinically significant prostate cancer, we observed an increase in its incidence between 2012 and 2022. Risk classification analysis showed a significant shift in the distribution of patients over the decade (*p* < 0.001, Table 1). The proportion of low-risk cases decreased from 10% in 2012 to 3% in 2022, while high-risk cases increased from 30 to 40% during the same period. The incidence of advanced cancer remained relatively stable at approximately 20%. These trends suggest a more selective diagnosis toward clinically significant tumors, effectively reducing the identification of indolent cases.

### Trends in rationale for PSA measurements

Approximately 40% of PSA measurements were performed in general internal medicine clinics, and around 30% in urology clinics, with this distribution remaining stable over time. Prostate cancer was detected through repeat measurement due to previously elevated PSA (PSA monitoring) in 13–17% of cases. Consistent with the discontinuation of organized screening in Shiga Prefecture, the proportion of patients diagnosed through this method was extremely low (2% in 2012, 3% in 2017) and reached 0% in 2022. While this small subset represents the residual impact of previous screening programs, the overall trend confirms that prostate cancer detection in this region has shifted almost entirely to opportunistic clinical practice. The proportion of cancers identified during health check-ups increased from 9% in 2012 to 14% in 2022, possibly reflecting the migration of men previously screened via organized programs into the comprehensive health check-up group. The proportion of prostate cancers diagnosed during evaluation for metastatic disease of unknown origin remained consistent at 4% across all years. Overall, there were no significant changes in the reasons for PSA measurement over time (Table 2).

**Table 2:** Reasons for PSA measurements.

Year	2012	2017	2022
Total	431 (100%)	553 (100%)	732 (100%)
General practice clinic	178 (41%)	210 (38%)	281 (38%)
Urologic clinic	125 (29%)	151 (27%)	196 (27%)
Repeat measurement due to previously elevated PSA (PSA monitoring)	57 (13%)	95 (17%)	107 (15%)
Organized screening (population-based screening)	10 (2%)	19 (3%)	0
Health check-up (active measurement)	37 (9%)	50 (9%)	100 (14%)
Evaluation for metastatic disease of unknown origin	18 (4%)	20 (4%)	31 (4%)
Others	6 (1%)	8 (1%)	17 (2%)

### Selections of primary treatment

**Fig. 1:**
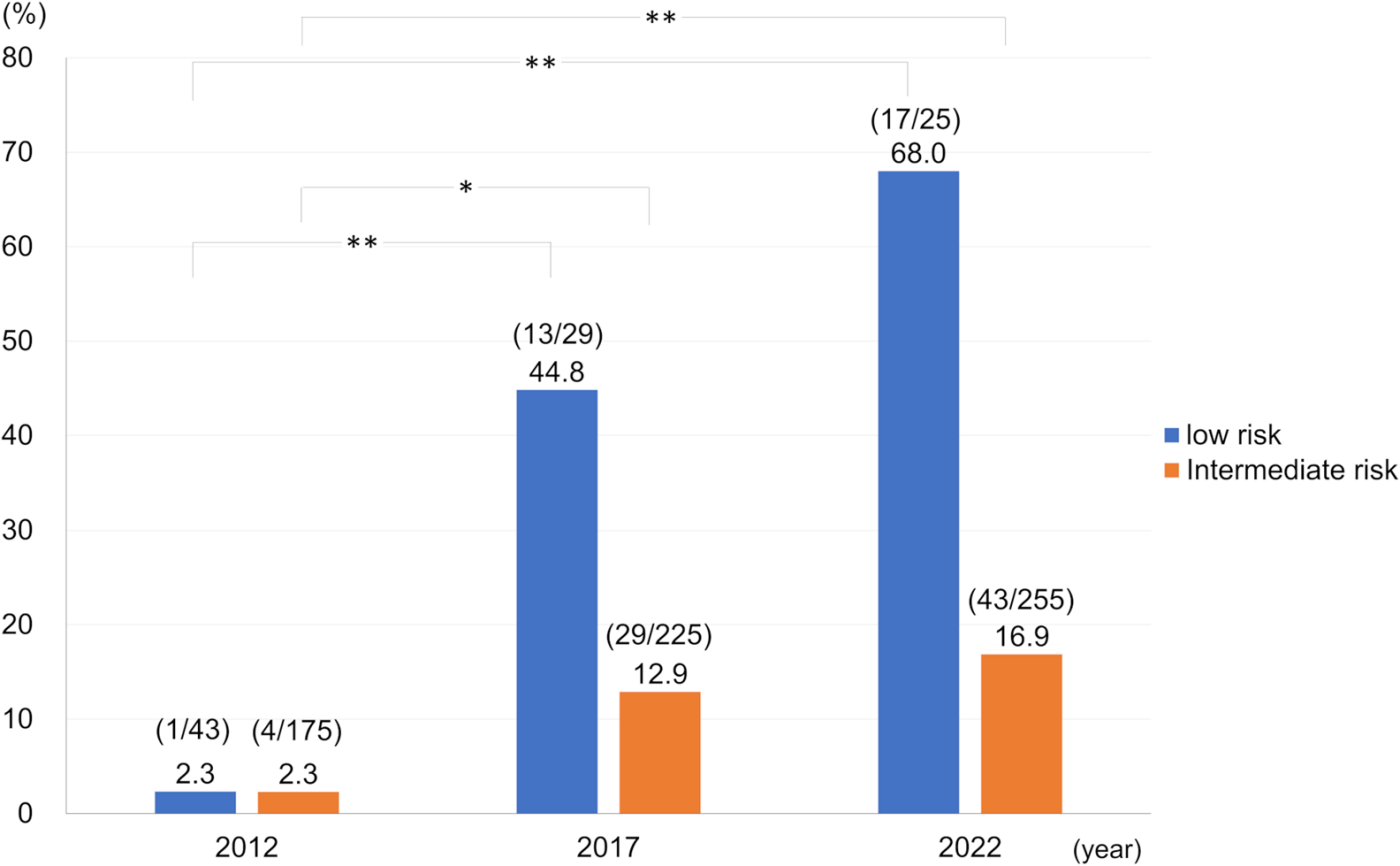
Changes in the proportion of low- and intermediate-risk prostate cancer patients selecting active surveillance. *p < 0.01, **p < 0.001.

The proportion of patients undergoing radical prostatectomy as initial treatment remained stable at approximately 25% across survey years. The use of radiotherapy or hormone therapy decreased slightly in 2022 compared with 2012. In contrast, the adoption of active surveillance (AS) increased markedly, from roughly 1% of patients in 2012 to 8% in 2017 and 9% in 2022 (Table 3). Notably, among low-risk patients, the proportion opting for AS rose significantly from 2.3% in 2012 to 68% in 2022, while in the intermediate-risk group, it increased from 2.3 to 16.9% over the same period (Fig. 1). The combination of fewer low-risk diagnoses and increased AS indicates that unnecessary invasive intervention for clinically insignificant cancer is being successfully minimized.

**Table 3:** Primary treatment for prostate cancer in study population.

Year	2012	2017	2022
Total	431 (100%)	553 (100%)	732 (100%)
Radical prostatectomy	107 (25%)	143 (26%)	174 (24%)
Radiation therapy	166 (39%)	179 (33%)	250 (34%)
Hormonal therapy	141 (33%)	175 (32%)	217 (30%)
Active surveillance	5 (1%)	45 (8%)	67 (9%)
Others	12 (3%)	11 (2%)	24 (3%)

### Proportion of patients with advanced prostate cancer separated by the reason for undergoing PSA evaluation

**Fig. 2:**
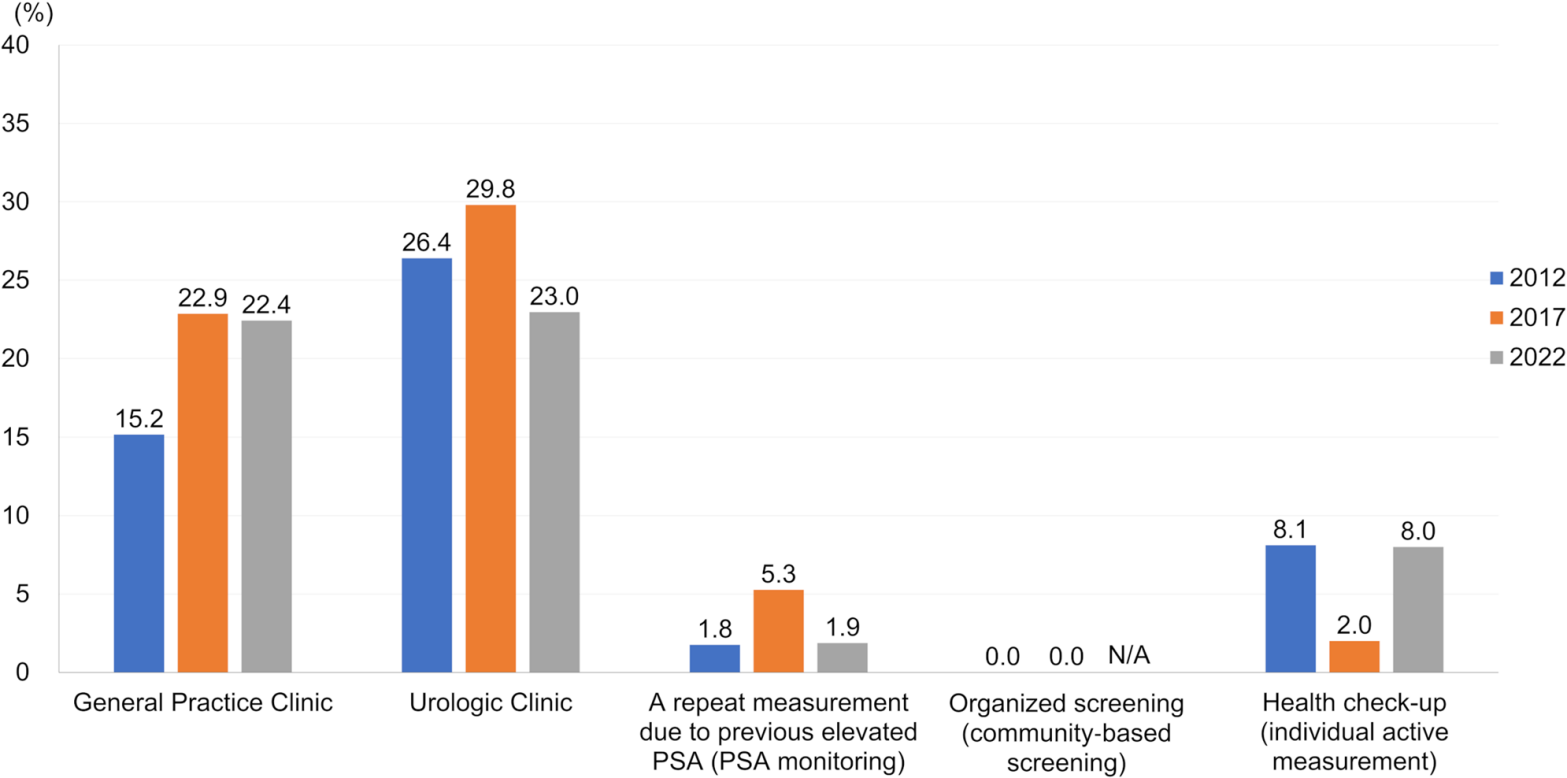
Proportion of patients with advanced prostate cancer according to the reason for PSA testing.

The relationship between the reason for PSA testing and the proportion of patients with advanced prostate cancer is shown in Fig. 2. The highest proportion of advanced cancers was observed among patients tested in urology clinics (23.0–29.8%), followed by those tested in general internal medicine clinics (15.2–22.9%). As shown in Fig. 2, these opportunistic testing groups—likely representing men with a limited history of PSA testing—exhibited a higher prevalence of advanced prostate cancer in our cohort. In contrast, the rate of advanced cancer detected through organized screening was 0% among the small number of cases identified through municipal programs in 2012 and 2017. Among patients undergoing PSA monitoring, the rate of advanced disease was low (1.9–5.3%), and similarly, in individuals who received PSA testing during health check-ups, the proportion of advanced cancer remained low (2.0–8.0%).

## Discussion

This study provides a longitudinal assessment of the current state of prostate cancer diagnosis and treatment in a Japanese prefecture characterized by the absence of an organized, systematic screening program. While population-based screening is not implemented in this region, PSA testing is performed opportunistically within routine clinical practice. This unique environment allows for the characterization of prostate cancer trends without the influence of systematic intervention. Our findings indicate that opportunistic PSA testing in routine clinical practice has not improved early detection over the past decade. Despite clinical advancements, such as the widespread use of PSA testing in general practice clinics, the prevalence of M1-stage (11–15%) and advanced (19–21%) cancers in our cohort remained relatively high.

These findings are consistent with recent reports from other Japanese prefectures^27,28^. For instance, a study from Niigata reported that the proportion of metastatic prostate cancer was 4.5% in the population based-screening group^27^. However, based on their data, the rate for cases identified in the non-screening group is calculated to be 22.6%. Moreover, a 15-year analysis in Yokosuka demonstrated a stark contrast in the advanced rate between clinically detected cases (14.97%) and those detected through organized screening (5.22%). Crucially, the Yokosuka data also revealed that the screening-detected group had significantly better overall and cancer-specific survival^28^. While a comprehensive evaluation of screening requires consideration of potential psychological and physical harms associated with the procedure, these data underscore that cancers detected through organized screening are consistently identified at a less advanced stage.

In our cohort, the prevalence of advanced prostate cancer among individuals undergoing PSA monitoring and health check-ups was consistently low (1.9–8%), which is highly comparable to the 3.7% reported for opportunistic screenings in the Niigata study. These findings suggest that men who are more proactive about their health are more likely to request PSA measurements, thereby reducing the incidence of advanced and metastatic prostate cancer at the time of diagnosis. As reported by Okihara et al.^29^, a systematic screening program reflecting the growing concern over prostate cancer effectively increased screening uptake among residents, leading to a reduction in metastatic cancer. While the current study focuses on a single region without a direct comparison group, our findings imply that the lack of a systematic framework may result in lower public awareness, leaving many high-risk individuals unscreened until their cancer has progressed.

The present study demonstrated a decline in the diagnosis of clinically insignificant prostate cancers over the past decade. This trend aligns with recent reports from other regions, such as Australia, where a reduction in GG1 diagnoses was partly attributed to the increasing use of pre-biopsy MRI^30^. Although specific data on MRI utilization were not available in our database, the widespread adoption of image-guided diagnostic pathways, as supported by the PRostate Evaluation for Clinically Important Disease: Sampling Using Image-guidance Or Not? (PRECISION) study^25^, may have contributed to the more selective detection of clinically significant disease in our clinical practice. Furthermore, while emerging biomarkers such as the Prostate Health Index or α2,3-sialylated PSA%, show promise in further reducing unnecessary biopsies^31–33^, our findings suggest that contemporary diagnostic approaches may have the potential to reduce the detection of clinically insignificant cancer previously associated with PSA testing.

This evolution in clinical practice is reflected in the significant increase in active surveillance for low-risk cases observed in our cohort. We found that the proportion of low-risk patients opting for AS rose to approximately 70% by 2022, a trend consistent with recent large-scale analyses in the United States and Japan^34,35^. Specifically, a recent nationwide multi-institutional study in Japan reported a marked shift toward conservative management for low-risk cases, reflecting changes in initial treatment patterns^35^. The safety and efficacy of this strategy are supported by landmark trials such as the randomized and controlled Prostate Cancer Intervention Versus Observation Trial (PIVOT), the Prostate Testing for Cancer and Treatment (ProtecT) trial, and the Prostate Cancer Research International Active Surveillance (PRIAS) study, which collectively demonstrate that AS or observation for early-stage, low-risk prostate cancer does not compromise long-term cancer-specific mortality compared with immediate radical treatment, while allowing a substantial proportion of patients to avoid the morbidity of local treatment^36–38^. Although these trials do not include untreated control groups and thus do not permit definitive conclusions regarding the absolute clinical benefit of screening itself, they provide a clinical basis for the use of AS in risk-stratified management. Collectively, our results suggest that clinical practice in Shiga has evolved toward more appropriate, guideline-based management, effectively reducing the burden of unnecessary invasive intervention especially for low- and intermediate-risk prostate cancer.

From an epidemiological perspective, the lack of demonstrated overall survival (OS) benefit in large-scale randomized trials remains a significant point of contention. The US Preventive Services Task Force (USPSTF) recommends against screening for men aged 70 and older, as potential harms outweigh the benefits^39^. Our finding that the median age at diagnosis in Shiga has increased to 74 years further underscores the complexity of applying these guidelines in an opportunistic clinical setting. Furthermore, as recently scrutinized by Lucassen et al.^40^, the feasibility of detecting more cancers through advanced diagnostic tools does not equate to improved mortality or quality of life. They emphasize that increased detection may merely amplify overdiagnosis without addressing overall clinical benefit. In this context, the ethical implications of opportunistic testing in healthy individuals—particularly in the absence of formal shared decision-making processes—have been questioned in relation to the principles of the Helsinki Declaration. Our study, being a descriptive analysis, does not attempt to refute these concerns or validate the efficacy of screening. Instead, it provides an objective, real-world report on how prostate cancer is managed, reflecting the challenges of regional healthcare regardless of the ongoing debate over screening benefits. While our study does not allow for a direct assessment of screening efficacy, these findings suggest the need for balanced information delivery, particularly to unscreened populations, to optimize the quality of opportunistic testing.

This study has several limitations. First, the data were obtained from a limited geographic area of Japan and may not be representative of the nationwide population. Second, interobserver variability may exist in the pathological assessments performed across institutions. Third, differences in diagnosis and treatment could reflect the specific practice policies of individual attending physicians. Fourth, as a descriptive analysis of patients already diagnosed with prostate cancer, this study does not evaluate the overall effectiveness of population-based PSA screening or its impact on mortality. Despite these limitations, our 10-year longitudinal data provide important insights into the challenges of pursuing early detection within an opportunistic testing environment.

In conclusion, our analysis of three distinct time points over a decade revealed that the stage and grade of prostate cancer at diagnosis did not improve within this opportunistic testing environment. While clinical practices have evolved to successfully minimize unnecessary invasive intervention, these findings suggest that clinical progress alone cannot fully compensate for the lack of organized efforts to improve early detection. Crucially, this study provides unique and essential insights into the natural trend of prostate cancer diagnosis and management in a real-world setting specifically characterized by the absence of organized screening.

## Data Availability

Raw data were generated at Shiga University of Medical Science. Derived data supporting the findings of this study are available from the corresponding author A.W. on reasonable request.

## Abbreviations

PSA: Prostate-specific antigen
ERSPC: European randomized study of screening for prostate cancer
PLCO: Prostate, lung, colorectal, and ovarian
CAP: The cluster randomized trial of PSA testing for prostate cancer
ISUP: International society of urological pathology
GG: Grade group
AS: Active surveillance
MRI: Magnetic resonance imaging
USPSTF: United States preventive services task force
